# Development of a Three-Dimensional (3D) Printed Biodegradable Cage to Convert Morselized Corticocancellous Bone Chips into a Structured Cortical Bone Graft

**DOI:** 10.3390/ijms17040595

**Published:** 2016-04-20

**Authors:** Ying-Chao Chou, Demei Lee, Tzu-Min Chang, Yung-Heng Hsu, Yi-Hsun Yu, Shih-Jung Liu, Steve Wen-Neng Ueng

**Affiliations:** 1 Department of Mechanical Engineering, Chang Gung University, Taoyuan 33302, Taiwan; enjoycu@ms22.hinet.net (Y.-C.C.); dmlee@mail.cgu.edu.tw (D.L.); s0937277402@gmail.com (T.-M.C.); hsuyh@adm.cgmh.org.tw (Y.-H.H.); alanyu1007@gmail.com (Y.-H.Y.); 2 Department of Orthopedics, Chang Gung Memorial Hospital, Taoyuan 33375, Taiwan; wenneng@cgmh.org.tw

**Keywords:** biodegradable bone cage, 3D printing, polylactide (PLA), corticocancellous chips, structured strut bone graft

## Abstract

This study aimed to develop a new biodegradable polymeric cage to convert corticocancellous bone chips into a structured strut graft for treating segmental bone defects. A total of 24 adult New Zealand white rabbits underwent a left femoral segmental bone defect creation. Twelve rabbits in group A underwent three-dimensional (3D) printed cage insertion, corticocancellous chips implantation, and Kirschner-wire (K-wire) fixation, while the other 12 rabbits in group B received bone chips implantation and K-wire fixation only. All rabbits received a one-week activity assessment and the initial image study at postoperative 1 week. The final image study was repeated at postoperative 12 or 24 weeks before the rabbit scarification procedure on schedule. After the animals were sacrificed, both femurs of all the rabbits were prepared for leg length ratios and 3-point bending tests. The rabbits in group A showed an increase of activities during the first week postoperatively and decreased anterior cortical disruptions in the postoperative image assessments. Additionally, higher leg length ratios and 3-point bending strengths demonstrated improved final bony ingrowths within the bone defects for rabbits in group A. In conclusion, through this bone graft converting technique, orthopedic surgeons can treat segmental bone defects by using bone chips but with imitate characters of structured cortical bone graft.

## 1. Introduction

Over the past decades, large segmental bone defects caused by trauma, infection, bone tumor, or revision surgeries have represented big challenges for orthopedic surgeons [1]. Bone defect repair has been a topic of common interest for the clinical, biological, materials science, and tissue engineering research areas [2]. When treating bone defects, one of the main requirements is the need of adequate bone grafts to fill these defects. Various graft types are used to fulfill the bone defect, such as autogenic, allogenic, or synthetic bone materials of various sizes and shapes [3,4]. For the bone grafting procedure to be successful, numerous novel implants also have been designed to provide durable stability around the bone defect site, which including fixed-angle locking plates or intramedullary locking nails with multiple locked screw designs [5,6,7].

Although a multitude of bone defects respond well to the current treatment strategies, challenges and limitations still exist due to the use of bulky implants, which include soft tissue envelope compromise and progressively bony deformities during treating tibia plateau distal femoral, or proximal humeral fractures [8,9,10]. For these reasons, there is an imperious need for bone grafts that can provide both biologic bone healing capacities and mechanical bony supports to decrease the requirement of those bulky implants. In such cases, structural bone grafts like fibular or tri-cortical strut iliac bone grafts are recommended for some bone defect management, especially for those patients with osteoporotic problems [11].

When using bone grafts, one major limitation is the limited supply, especially cortical structured bone grafts, no matter from autogenic or allogenic resources. Therefore, it is a superior concept to find some biocompatible materials that can sufficiently fulfill bone defects, provide adequate mechanical supports, and effectively promote rapid bone formation [12,13]. Polylactide (PLA) is one of the most promising biopolymers. It is known to decompose easily by simple hydrolysis of the ester backbone, resulting in the formation of non-harmful and non-toxic monomers. Owing to their excellent biocompatibility and mechanical properties, PLA and its copolymers have been extensively used in tissue engineering, drug delivery, and medical implants manufacturing [14,15].

Three-dimensional (3D) printing, also known as additive manufacturing, is a novel procedure that is used to create three-dimensional objects [16]. Among the different technology options, 3D printing is becoming popular due to its capacity to directly print porous scaffolds with a designed shape, controlled chemistry, and interconnected porosity [17]. The use of 3D printing technologies in bone tissue engineering has been growing in recent years [18,19]. In the present study, we designed and developed a three-dimensional printed PLA cage, filled with corticocancellous bone chips, to serve as a structured bone graft. We hypothesized that by using this 3D printed cage, we will be able to successfully convert morselized corticocancellous bone chips into a structured bone graft to treat the bone defect in a rabbit segmental femoral model.

## 2. Results

**Figure 1:**
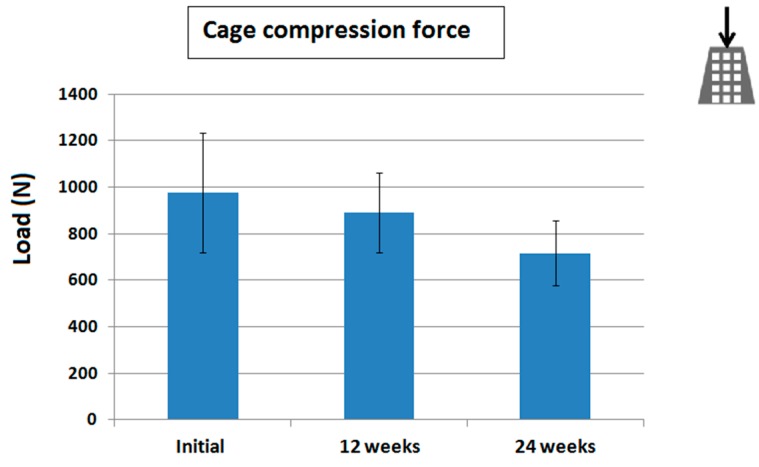
The three-dimensional (3D) printed cage degradation test. Compressive results showed averaged maximal compressive loads of 977, 889, and 715 N immediately after, at 12 and 24 weeks after immersion in phosphate buffer solution (PBS), respectively. These results evidenced the cage degradation. Photography on right upper corner represented the direction of the compression test.

The biodegradable bone cage was effectively fabricated using the 3D printer. The average compression strength of the printed cage was 977 N initially. Following the degradation test, the compression strength decreased to 889 and 715 N (9.0% and 26.8%) after 12 and 24 weeks of immersion in phosphate buffer solution (PBS), respectively (Figure 1).

All 24 rabbits underwent the surgical procedure smoothly and no intraoperative animal death was reported. We found no surgical wound breakdown, hematoma formations, deep wound infection, or Kirschner-wire (K-wire) penetration from the knee wound. All rabbits consumed an increasing amount of chow diet and water during the 3 days following surgery and devoured stationary over the following 2-week period. No significant difference was observed when comparing the volumes consumed by the two groups of animals. Furthermore, all animals completed their postoperative care course uneventfully, without death, before sacrifice.

### 2.1. Animal Activity

**Figure 2:**
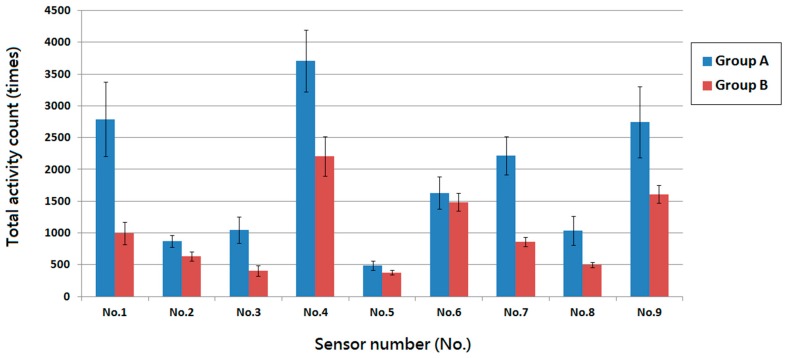
The postoperative one-week total activity counts of rabbits in the Animal behavior cage. Rabbits in group A showed higher total activity counts compared to that of rabbits in group B. Rabbits in both groups showed relatively higher numbers of triggers at sensor locations No. 4 and No. 6 which indicated more frequent visits to the food and water supply site. Rabbits in group A also demonstrated higher numbers of triggers at sensor locations No. 1, No. 7 and No. 9 but less at senor location No. 5, which represented rabbits were addicted to stay at the corners and avoided to stay in the center of the cage.

Figure 2 shows the two groups’ activity counts over the 1-week postoperatively period. The counts obtained from the photoelectric switch sensors were 16,954 ± 2466, and 9220 ± 1516 for groups A and B, respectively. Additionally, rabbits in group A demonstrated higher numbers of triggers at sensor locations No. 1, No. 7, and No. 9. This indicated more frequent position changes between the corners of the cage. The same trend was observed at sensor location No. 4, accounting for increased number of visits to the food supply site.

### 2.2. Radiographic and Histological Examination of Bone Healing

**Figure 3:**
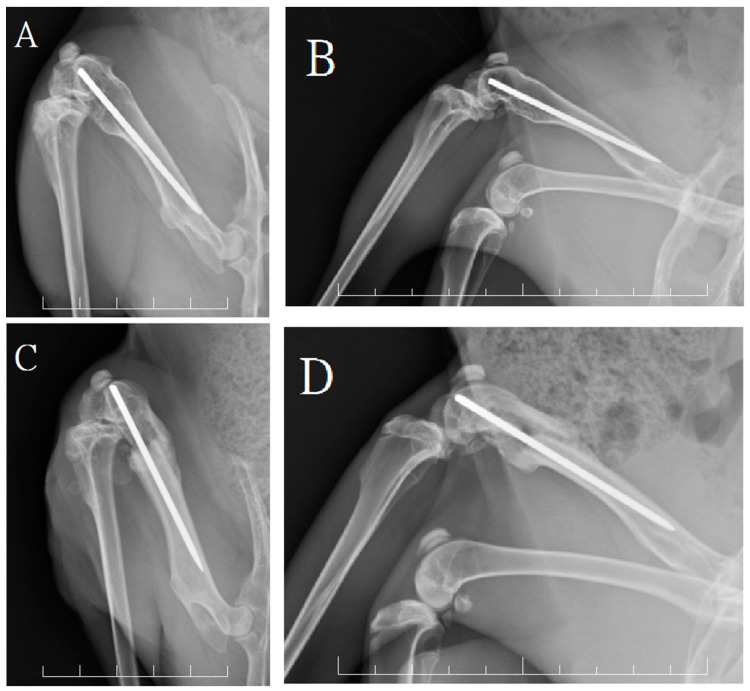
The radiographic assessments at 24 weeks. The anterioposterior (**A**) and lateral (**B**) view of a rabbit in group A showed good bone growth within the bone defect site with intact anterior cortical integrity; The anterioposterior (**C**) and lateral (**D**) view of a rabbit in group B showed callus formation but had broken of the anterior cortex. Each frame rates one centimeter.

The radiographic examinations results at one week postoperatively evidenced that 16.6% of rabbits in group A (2/12) and 91.6% of rabbits in group B (11/12) exhibited breaking of the residual anterior femoral cortical bones, with a significant difference between the two groups (*p* < 0.05). The 6-weeks postoperative radiographs for group B evidenced fracture site collapse with shortening of the bone defect gap, while the bones of the rabbits in group A showed no significant leg deformity. The radiographs of both groups at 12 or 24 weeks postoperatively showed good bridging callus formation around the bone defect site without residual cortical bone defects or nonunion formation (Figure 3).

**Figure 4:**
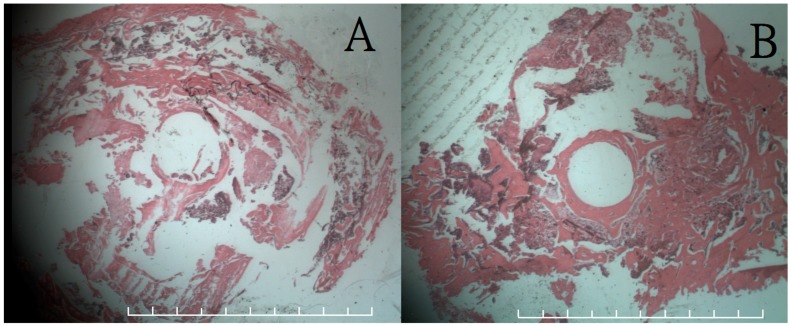
Histological results (200×) of coronal section on femoral bone defect site with hematoxylin and eosion (H & A) stain. (**A**) The results at 12 weeks showed no inflammatory overreaction around the inferior aspect of the defect site; (**B**) The results at 24 weeks revealed good bone callus formation around the entire defect site. Each frame rates one millimeter.

Figure 4 shows coronal sections of the histological examination at 12 and 24 weeks postoperatively of femoral sample from rabbits in group A. The histological image at 12 weeks showed no inflammatory cells overreaction around the inferior aspect of the defect site, while the image at 24 weeks revealed good bone callus formation around the whole defect site. No residual 3D printed cages can be recognized in both 12- and 24-week sectional specimens. It was due to the dissolving effect of formic acid solutions during the decalcification procedure of the sectional slide production.

### 2.3. Leg Length and Mechanical Strength Test

**Figure 5:**
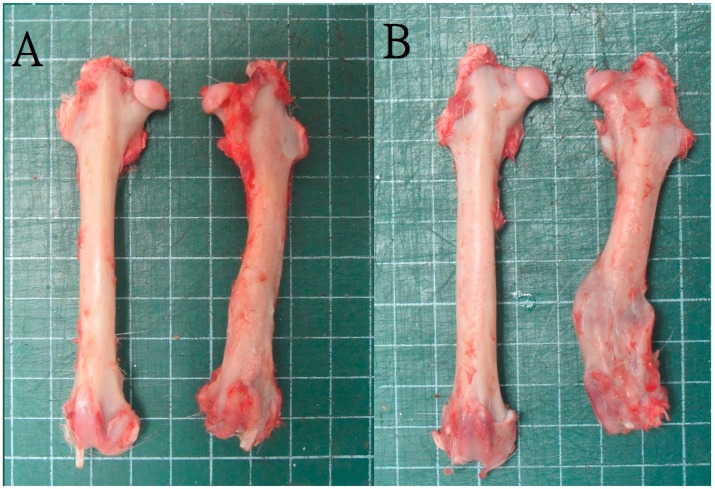
Leg specimens for leg-length ratio study. (**A**) A rabbit in group A showed good bone healing with conservation of femoral length; (**B**) A rabbit in group B showed plenty bridging callus formation but shortening of left femoral length.

**Figure 6:**
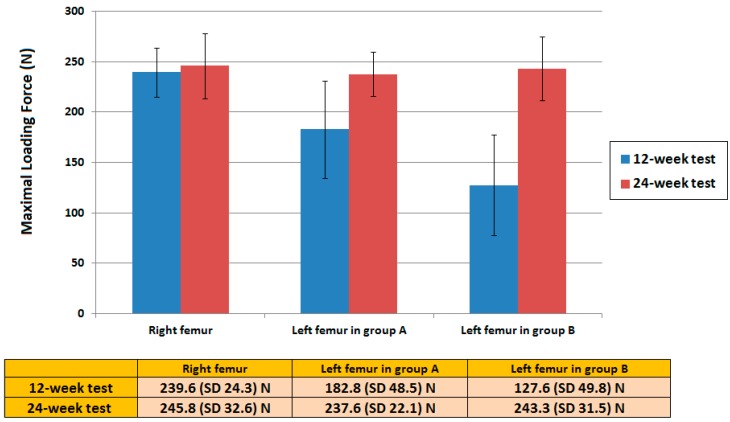
The 3-point bending strength test. The bending strength of the left femur in group A at the 12-week test revealed no statistic difference than that of the healthy leg. However, the strength of the left femur in group B was considerably smaller than that of the right healthy leg. In the 24-week test, the bending strengths of the left femur in both groups showed no significant difference than that of the right femur.

The leg length comparison was conducted following animal sacrifice for all the 24 rabbits. The leg length ratio showed a significant difference between the two groups: 94% (average, 9.6/10.2 cm) in group A and 81% (average, 8.25/10.2 cm) in group B (Figure 5). The maximal bending strength of the 3-point bending test for the left femur in group A at postoperative 3 months revealed a slightly lower than that of the healthy leg but with no statistic difference. Nevertheless, the bending strength of the left femur in group B was considerably smaller than that of the right healthy leg. There was also a statistic different strength of left femur between group A and group B. At the postoperative 6-month test, the bending strengths of the left femur in both groups showed no significant difference than that of the right femur, which represent adequate bone healing of the left femoral bone defect site on both groups at this time interval (Figure 6).

## 3. Discussion

This experimental study aimed to develop a new cage using synthetic biodegradable materials by converting corticocancellous bone chips into a structured strut bone graft. This cage could be used for the treatment of segmental bone defects. In the animal femoral bone defect model, the use of an intramedullary K-wire fixation only provided the rabbit with an acceptable protection of the bony alignment, but did not provide sufficient maintenance of the leg length and rotational stability. In this study, the special bone defect design that included preserving the anterior bony cortex could help assess any further bony collapse or deformity after the index operation. The 3D printed cage developed in this study facilitated fracture fixation and enhanced bone healing by providing fragment-seizing and mechanical supporting characteristics. The experimental results revealed an increased number of total activity counts one week postoperatively for the rabbits that received the 3D printed cage when compared to those without the cage. These results indicated better bony fragments stabilization with less fracture pain due to fragmental irritations of the periosteum [20]. The imaging examination results showed less anterior cortical bone breakage one week postoperatively.

Autogenous bone grafts, the standard principle for bone defect treatments, provide several advantages, including low number of disease transmission complications and increased osteogenic capacities [3,4,21,22]. However, autogenous bone grafts may not always be available because of limited donor sources and large number of bone defects. Furthermore, when a large autogenic cortical structured bone graft is required to fill a considerable bone defect and provide mechanical support, patients generally experience an increased level of pain or functional donor site morbidities [23,24]. Given these concerns, allograft bone transplantation has developed rapidly and became a popular treatment option. For example, literatures report the use of fibular allografts to augment proximal humeral fractures [25,26]. The healing process following the allograft transplantation involves bone allograft revascularization, new bone formation, and union between the host’s bone bed and the graft [27]. Radiological and histological studies of large bone allograft confirmed that the repair process of the cortical bone allograft was extremely slow [28,29]. Up to date, the healing rates of segmental bone defect treated by biomaterials in animal studies are variable due to different bone defect models, unlike biomaterial shapes, and dissimilar animal selections [18,19,22,30]. On the other hand, the bone healing rate comparisons between biomaterials and allograft are also rare because no clinical study available on human of bone defect treated by biomaterials only and less animal model treated by allograft due to rare of animal bone bank setting up. In this direction, the results of the present study offer an alternative method. Using the 3D printing technique, orthopedic surgeons will be able to design and develop a cage of any required geometry to fit the entire bone defect while offering suitable mechanical support. The printed cage could be filled with the autogenic cancellous or corticocancellous bone chips. Further, orthopedic surgeons can use the composite cage as a cortical strut graft to adequately fill the entire bone defect while availing the mechanical properties for segmental bone defect management. The 3D printing technique is a novel process used to make a 3D object. During 3D printing, additives are used, in which successive layers of PLA material are laid down under computer control. Objects to be printed out can be of almost any shape or geometry, and can be produced from a 3D model or other electronic data source [30]. Owing to the successful outcome of this study, the 3D printing technique might be easily applied for the treatment of bone defects. PLA is thermoplastic and biodegradable aliphatic polyester obtained from naturally occurring organic acid (lactic acid) [31,32,33,34]. It has a glass transition temperature of 60–65 °C, a melt temperature of 180–220 °C, and degrades over a time period of about two years [31]. During the 3D printing process, the PLA filament was heated between its glass transition and melt temperature for winding and unreeling purposes. The *in vitro* degradation test showed that after 6 months of immersion in PBS the 3D cage maintained its high compressive strength. Therefore, the 3D printed cage can provide sustained mechanical support for bone graft consolidation. The *in vivo* animal study and the histological assessment results also showed good biodegradability and biocompatibility by providing a smooth bone healing process accompanied by no local or systemic tissue overreactions and successful bone growth 24 weeks postoperatively in all the rabbits used for the study.

Despite of the proven effectiveness of the 3D printed cage to convert bone chips into a strut bone graft on the rabbit femoral model, this study had some limitations. The first limitation is that we first proposed and manufactured the 3D cage, followed by the creation of the bone defect on the rabbit’s femur to fit this 3D cage. Even if these are not the actual clinical sequences in fracture management, this was the only way we could effectively fill the bone defect and quantitatively assess the leg length discrepancy. The second limitation is related to the metal implant design. The intramedullary K-wire fixation on the femoral site proved a good choice to maintain the bony alignment and allowed sliding compression of the femoral fragments especially after anterior cortex fracture. Once the anterior cortex is broken, the sliding collapse of the bone defect would enhance fragmental impactions within the bone defect and boost the corticocancellous chips to heal. This might explain why all femoral defects healed without nonunion and no fibrous tissue invasion phenomenon around the defect gaps was observed. Further scientific studies would be needed to investigate a more durable and stable implant fixation. Such future studies should concentrate on preserving the bone length and accentuate the different properties of the proposed cage. The third limitation of this study is the relatively restrictive sample size of 6 animals in each group for the histological assessments. Most prospective studies have used a study population of at least 15 per group [35]. As we have only one animal behavior cage for assessing the one week rabbit activity after operation, and in order to complete the animal sacrifice and mechanical evaluation on time, we can only have 12 rabbits in each study group for clinical comparisons. More animals will be needed for the future studies.

We hope that eventually the biodegradable 3D printed cage, in combination with any corticocancellous bone chips, might eliminate the need for autogenous cortical bone grafting and decrease the number of problems associated with donor site morbidity. Furthermore, to address the donor source shortage, one can use autogenic, allogenic, and synthetic bone materials altogether to pad the biodegradable cage. This can fill the defect and overcome any cortical donor site restriction or bone bank reduced supply.

## 4. Experimental Section

### 4.1. Biodegradable Bone Cages

#### 4.1.1. Fabrication of the Three-Dimensional (3D) Printed Bone Cage

**Figure 7:**
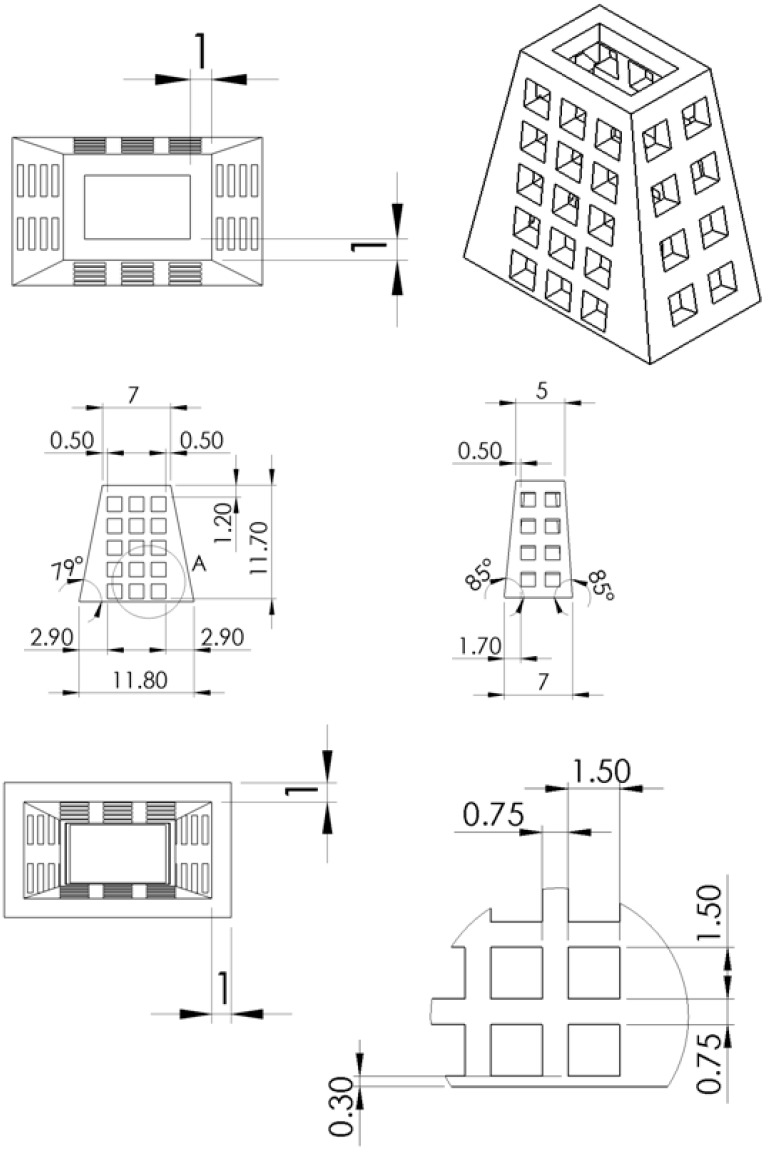
The design and dimension of the 3D printed truncated-pyramid cage.

A biodegradable truncated-pyramid cage was designed and manufactured to serve as the bone graft. Commercially available PLA filaments (Prolink Microsystems Corp., Taipei, Taiwan) with a diameter of 3.0 mm were used as the material for the bone cage 3D printing. Figure 7 shows the layout and dimensions of the cage. The cage was fabricated using a fused-deposition-modeling (FDM) type 3D printer (U-Maker, Taipei, Taiwan) with a printing resolution of 200 mm. During the printing procedure, the printer extruded small beads of material, which hardened immediately to form layers. The PLA filament that is wound on a coil was unreeled to supply material to the extrusion nozzle head. The nozzle head heated the material and turned the flow on and off. Two stepper motors were used to move the extrusion head and adjust the flow. A computer-aided manufacturing (CAM) software package (Solidworks, Waltham, MA, USA), was utilized to produce the code that was sent to a microcontroller that controls the motors. The maximum moving speed of the nozzle was 25 mm/second. The total time used to print a cage was approximately 20 min.

#### 4.1.2. Degradation and Mechanical Evaluation of the 3D Printed Cage

The molecular weight variation of PLA in the 3D printed cages was monitored by immersing the cages in a phosphate buffer solution (PBS) (0.15 mol/L, pH 7.4), 37 °C for 3 and 6 months, respectively. After their removal from the PBS and drying inside an oven for 24 h, the PLA cages molecular weights were measured by a gel permeation chromatograph equipped with a Waters 2414 Refractive Index Detector (Waters Corp., Milford, MA, USA).

The compression strengths of the 3D printed cage were evaluated using a Lloyd tensile tester (AMETEK, Largo, FL, USA) according to the ASTM D638 standard. The test used a 2.5 kN load cell and cross-head speed of 80 mm/min. The specimens (*n* = 5) were compressed along their axial direction and their maximum compression strengths were recorded.

### 4.2. Surgical Procedure and Animal Care

#### 4.2.1. Surgical Procedure

Twenty-four adult New Zealand white rabbits (Animal Health Research Institute, Panchiao, Taiwan) with a mean weight of 3.2 ± 0.9 kg were enrolled in this study. All animal-related procedures received the approval of Institutional Animal Care and Use Committee of Chang Gung University (IACUC Approval No:CGU14-093, Date of Approval: 1 December 2014), and all studied animals were cared for under the supervision of a licensed veterinarian in accordance with the regulations of the Department of Health and Welfare, Taipei, Taiwan.

**Figure 8:**
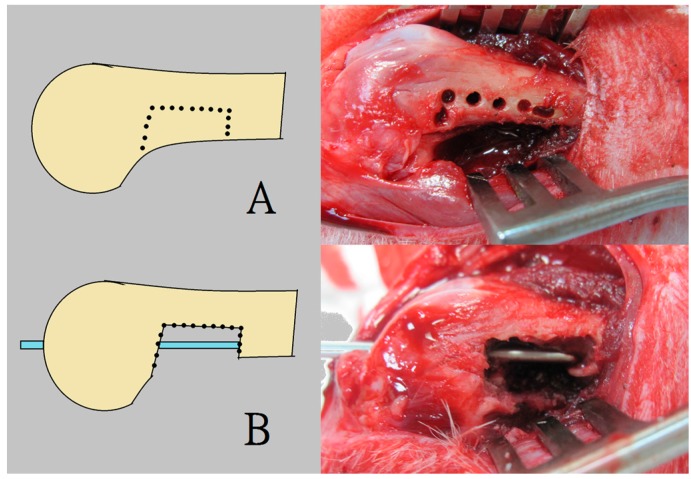
The surgical procedure for creating the rabbit femoral model of a segmental bone defect with intact anterior cortex. (**A**) The pre-drilling procedure was performed to create the bone defect; (**B**) Insertion of a 2 mm K-wire as an intramedullary metal implant fixation.

During the procedure, all 24 rabbits received general anesthesia through inhalation of isoflurane (Aesica Queenborough Limited, Queenborough, UK) in an anesthesia chamber comprised of a 40 × 20 × 28 cm transparent acrylic box. Following the anesthesia induction procedure, all rabbits were removed from the chamber. A sufficient anesthesia state was maintained during the entire surgical procedure using a mask that supplied a tolerable isoflurane level. Under satisfactory sterile conditions, all rabbits underwent a longitudinal incision of the left lateral thigh, followed by a periosteal dissection to expose the left femoral shaft. The pre-drilling procedure was performed using a 1.5 mm Kirschner-wire (K-wire) and an electrical-powered driller. This was done to create a bone defect territory of the same size as the 3D printed cage, on the inferior aspect of the femur, 1 cm proximal to the distal femoral condyle. Further, a tri-cortical bone defect was generated through the excision of the bone content within the pre-drilling site. The superior cortex of the femoral shaft on the bone defect site was kept intact without removal. Additionally, the surgical wound was extended into the left knee for the lateral parapatellar exposure of the distal femur. This procedure enabled drilling a hole in the intercondylar notch; the hole was used to insert a 2 mm K-wire that extended to the proximal end of the femur, thereby allowing the intramedullary metal implant fixation (Figure 8). The excised bone fragments were grinded into small pieces of less than 2 × 2 mm in size to serve as fillers for the corticocancellous bone grafts. The 3-D printed cage was immersed in 95% alcoholism for 5 min before the surgical application.

**Figure 9:**
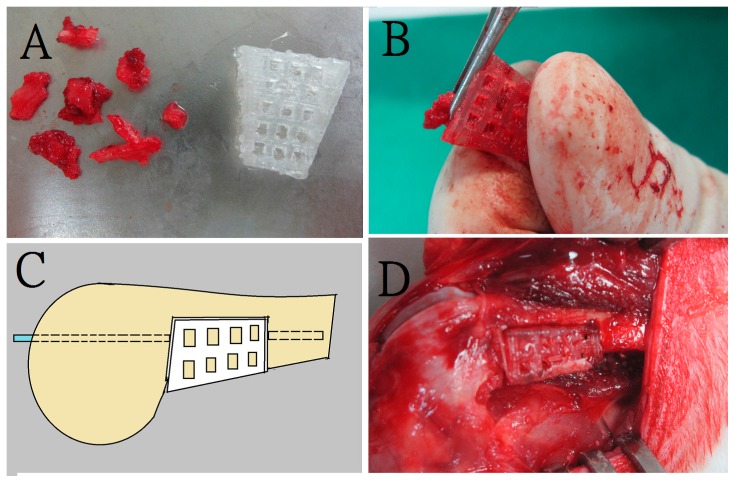
The bone grafting procedure in group A. A rabbit in group A underwent bone defect creation and insertion of a 3D printed cage filled with corticocancellous bone graft (**A**) Preparation of morselized corticocancellous bone chips (**B**) Padding bone chips into the cage; (**C**) Illustration of series connection of the composite strut graft with femoral shaft by the intramedullary K-wire; (**D**) Photograph of the composite strut graft after fixation.

**Figure 10:**
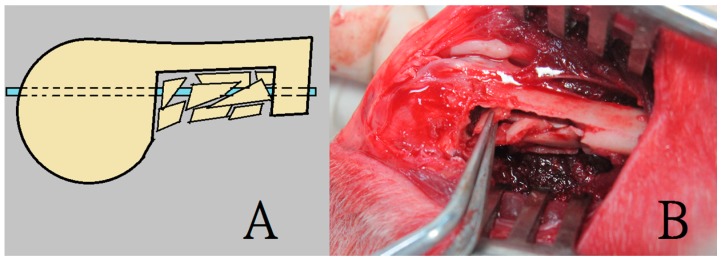
The bone grafting procedure in group B. A rabbit in group B sustained bone defect creation and insertion of corticocancellous bone graft only (**A**,**B**) Illustration and photograph of an intramedullary K-wire insertion with implantation of corticocancellous bone chips.

The rabbits were randomly divided into 2 groups, group A and group B. For all the 24 rabbits, a bone defect was created first, followed by an intramedullary K-wire fixation. To fill the created defect, the 12 rabbits in group A received a PLA cage with corticocancellous excised bone chips (Figure 9), while the 12 rabbits in group B (control group) received corticocancellous excised bone chips only, without a PLA cage (Figure 10).

#### 4.2.2. Postoperative Animal Care and Clinical Assessments

**Figure 11:**
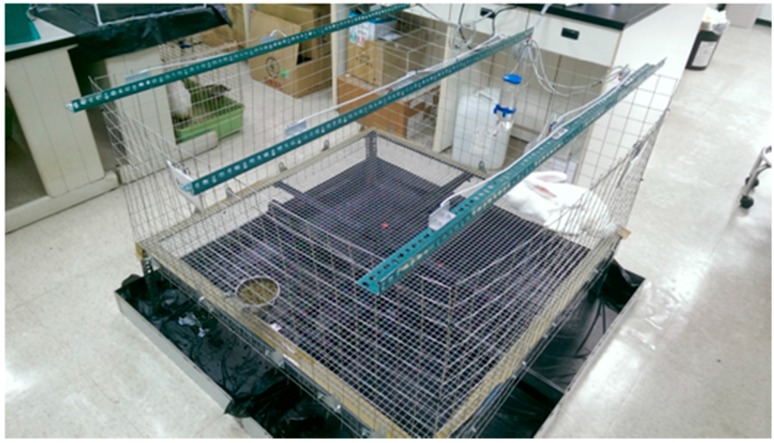
The animal behavior cage (ABC). The cage (1.2 × 1.2 × 0.7 m) consisted of a large floor and high walls.

**Figure 12:**
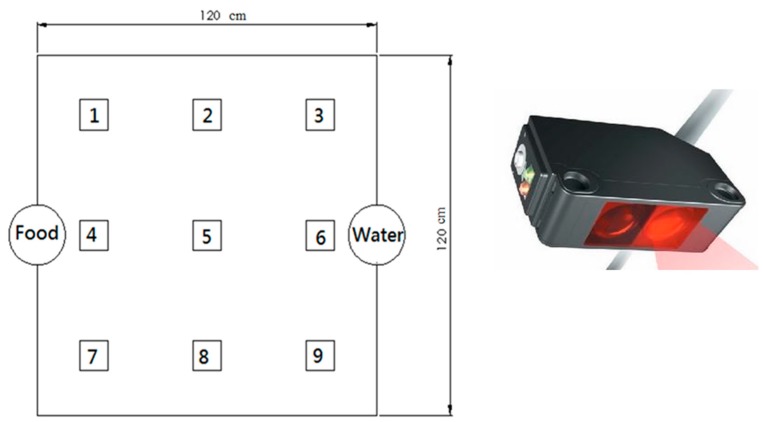
The basic distribution of switch sensors in the ABC. Each number in the cage represented the sensor number.

Following the surgical procedure, all rabbits were allowed to live freely, were given standard rabbit chow and sterilized drinking water *ad libitum.* The temperature and humidity of our institutional animal care center were maintained around 24 °C and 70%, respectively. The animals’ general activity was evaluated on a daily basis by housing the rabbit in a lab-made animal behavior cage (ABC) for one week, after which the rabbit was returned to its original individual cage for further animal care. As shown in Figure 11, the ABC cage (1.2 × 1.2 × 0.7 m) consisted of a generous floor and high walls. Nine diffusion-scan type photoelectric switch sensors, with a self-contained amplifier, (HP100-A1, Azbil Corp., Tokyo, Japan) were used to monitor the rabbit movement within the cage. The sensors were placed at the top of the cage, at a distance of 30 cm between one another. The sensors were clamped along the three bars separating the cage into nine symmetrical areas. (Figure 12) The sensors were adjusted to detect movement within a 250 × 250 mm volume of space around the base of the cage. As a rabbit moved from one area of the cage to another, the sensor in the “approaching” area would be triggered by the activity. The total number of triggers was recorded on a personal computer equipped with an acquisition interface. Each rabbit’s activity was monitored for one week.

All rabbits left thighs underwent radiologic examination one week postoperatively to evaluate the early fixation condition by assessing the anterior femoral cortical integrity. Furthermore, another set of radiologic examinations was conducted prior to sacrificing the animals (12 or 24 weeks) to assess the final bone healing conditions.

#### 4.2.3. Animal Sacrifice and Mechanical Evaluation

Following the imaging investigations, at 12 weeks postoperatively, six of the rabbits in each group were sacrificed by intravenous injections of 10 mL lidocaine. The remaining six rabbits from each group were sacrificed at 24 weeks postoperatively. Following this, mechanical evaluation and leg length comparisons were performed. The femoral specimens were dissected to gently remove the residual muscles and intramedullary K-wire. Both femurs (the left to be studied and the healthy right one) were harvested for femoral length comparisons. The leg length ratio was defined as the length of the index left femoral shaft divided by the length of the healthy right femoral shaft. For the mechanical evaluation, the proximal end of each femoral specimen was embedded it into an epoxy cylindrical block (radius = 1.5 cm, thickness = 1.3 cm). Thereafter, the specimens were secured onto the flexural test machine (QC-536, Cometech Testing Machines Co., Ltd., Taichung, Taiwan) for the 3-point bending strength assessment. The moving speed of the loading pin was set at 900 mm/min.

After the bending test, the broken left femoral specimen was prepared for histological examination. The specimen was decalcified by immersion in 5% formic acid for 3 days, dehydrated in a gradient ethanol series, embedded in paraffin, sectioned in 5 µm thickness slices, and stained with hematoxylin and eosin (H & E).

### 4.3. Statistics and Data Analysis

Data collected from quadruplicate samples were analyzed by one-way analysis of variance (ANOVA). Differences were considered statistically significant for *p* values <0.05.

## 5. Conclusions

The present experimental results indicate that using 3D printing PLA can be remodeled into a cage of any geometric shape to fill the bone. With this 3D printed cage, the orthopedic surgeons will be able to convert small corticocancellous bone chips into a structured bone graft to avail both the osteoconductive and mechanical properties. Through this bone graft converting technique, the surgeons can merge different sources of bone graft to serve as an adequate strut bone graft for the treatment of large bone defects without experiencing donor site morbidity and graft resource limitations.
